# School racial segregation and long-term cardiovascular health among Black adults in the US: A quasi-experimental study

**DOI:** 10.1371/journal.pmed.1004031

**Published:** 2022-06-21

**Authors:** Min Hee Kim, Gabriel L. Schwartz, Justin S. White, M. Maria Glymour, Sean F. Reardon, Kiarri N. Kershaw, Scarlett Lin Gomez, Daniel F. Collin, Pushkar P. Inamdar, Guangyi Wang, Rita Hamad

**Affiliations:** 1 Philip R. Lee Institute for Health Policy Studies, University of California San Francisco (UCSF), San Francisco, California, United States of America; 2 Department of Epidemiology & Biostatistics, UCSF, San Francisco, California, United States of America; 3 Stanford University, Stanford, California, United States of America; 4 Department of Preventive Medicine, Northwestern University Feinberg School of Medicine, Chicago, Illinois, United States of America; 5 Department of Family & Community Medicine, UCSF, San Francisco, California, United States of America; UCLA: University of California Los Angeles, UNITED STATES

## Abstract

**Background:**

Cardiovascular disease (CVD) disproportionately affects Black adults in the United States. This is increasingly acknowledged to be due to inequitable distribution of health-promoting resources. One potential contributor is inequities in educational opportunities, although it is unclear what aspects of education are most salient. School racial segregation may affect cardiovascular health by increasing stress, constraining socioeconomic opportunities, and altering health behaviors. We investigated the association between school segregation and Black adults’ CVD risk.

**Methods and findings:**

We leveraged a natural experiment created by quasi-random (i.e., arbitrary) timing of local court decisions since 1991 that released school districts from court-ordered desegregation. We used the Panel Study of Income Dynamics (PSID) (1991 to 2017), linked with district-level school segregation measures and desegregation court order status. The sample included 1,053 Black participants who ever resided in school districts that were under a court desegregation order in 1991. The exposure was mean school segregation during observed schooling years. Outcomes included several adult CVD risk factors and outcomes. We fitted standard ordinary least squares (OLS) multivariable linear regression models, then conducted instrumental variables (IV) analysis, using the proportion of schooling years spent in districts that had been released from court-ordered desegregation as an instrument. We adjusted for individual- and district-level preexposure confounders, birth year, and state fixed effects. In standard linear models, school segregation was associated with a lower probability of good self-rated health (−0.05 percentage points per SD of the segregation index; 95% CI: −0.08, −0.03; *p* < 0.001) and a higher probability of binge drinking (0.04 percentage points; 95% CI: 0.002, 0.07; *p* = 0.04) and heart disease (0.01 percentage points; 95% CI: 0.002, 0.15; *p* = 0.007). IV analyses also found that school segregation was associated with a lower probability of good self-rated health (−0.09 percentage points; 95% CI: −0.17, −0.02, *p* = 0.02) and a higher probability of binge drinking (0.17 percentage points; 95% CI: 0.04, 0.30, *p* = 0.008). For IV estimates, only binge drinking was robust to adjustments for multiple hypothesis testing. Limitations included self-reported outcomes and potential residual confounding and exposure misclassification.

**Conclusions:**

School segregation exposure in childhood may have longstanding impacts on Black adults’ cardiovascular health. Future research should replicate these analyses in larger samples and explore potential mechanisms. Given the recent rise in school segregation, this study has implications for policies and programs to address racial inequities in CVD.

## Abstract 2

Min Hee Kim and colleagues investigate the association between exposure to school racial segregation in childhood and long-term cardiovascular health among Black adults in the United States.

## Author summary

**Why was this study done?:**

- Inequitable access to educational opportunities among Black Americans may explain a disproportionate burden of cardiovascular disease (CVD) among this group, although it is unclear what aspects of education are most salient.
- School racial segregation, a manifestation of structural racism in the United States education system, may affect later cardiovascular health. Yet, few studies have rigorously examined the association between school segregation and long-term cardiovascular risk.

**What did the researchers do and find?:**

- We applied an instrumental variables (IV) approach leveraging quasi-random variation in the timing of court decisions since 1991 that released school districts from court-ordered desegregation.
- Increased school segregation was associated with poorer self-rated health and increased binge drinking among Black adults, and the latter was robust to adjustment for multiple hypothesis testing.

**What do these findings mean?:**

- Continuation of school segregation in the US may be a contributor to the high CVD burden in Black communities.
- National and local educational policies for school racial integration may help reduce the future burden of CVD among Black people.

## Introduction

Black people have the highest burden of cardiovascular disease (CVD) among all racial/ethnic groups in the United States [1,2], with CVD mortality 50% higher among Black people relative to White people [1]. This is mirrored by disparities in CVD risk factors during young adulthood, including higher rates of hypertension [3], physical inactivity [4], and binge drinking [5]. Educational attainment, which is strongly correlated with CVD, may play a role in producing these racial inequities [6,7]. Due to historical and ongoing structural racism, Black youth were 12 percentage points less likely to complete high school than White youth across the US in 2017 [8]. This matters for CVD, since educational attainment is associated with reduced risk of obesity and smoking—strong CVD risk factors—as well as mortality [9]. Furthermore, existing studies generally quantify education as years or credentials completed [9–11]; few measure differences in quality or other aspects of schooling [12,13].

School racial segregation is one major aspect of schools in the US whose health effects are less studied. Defined as the degree of physical separation of students in schools based on their race [14], school segregation remains a key feature of the US education system, in part because of recent “resegregation.” Black activists pushed for school racial desegregation (i.e., integration) during the 1950s to 1970s due to Black–White inequality in educational resources [15], and the Supreme Court’s landmark 1954 Brown v. Board of Education decision found that school racial segregation violated Black children’s constitutional rights. This resulted in school districts nationwide adopting court-ordered desegregation plans, and there was slow but substantial school racial integration throughout the US during the 1960s and 1970s [16–19]. The resulting desegregation improved Black Americans’ educational and occupational attainment, improved overall health [20,21], and may have reduced teen fertility [22,23]. These plans, however, were not intended to be permanent, and in 1991, the Supreme Court’s ruling in Board of Education v. Dowell and several subsequent cases made it easier for districts to be released from their desegregation orders [18]. Local court decisions have since released more than 600 of the approximately 1,000 school districts nationwide that had been under court-ordered desegregation, reverting many to neighborhood-based school assignment [16–19]. Levels of school racial segregation steadily grew in released districts, reversing decades of progress in school racial integration [17,18,24–26]. Nationally, highly segregated schools with less than 10% White enrollment have more than tripled in recent decades to nearly 20% of all schools [16]. While 1 study found this rise in school segregation was associated with a higher preterm birth rate among Black women [27], no other studies, to our knowledge, have examined the influence of recent trends in school resegregation on health among Black adults.

**Fig 1:**
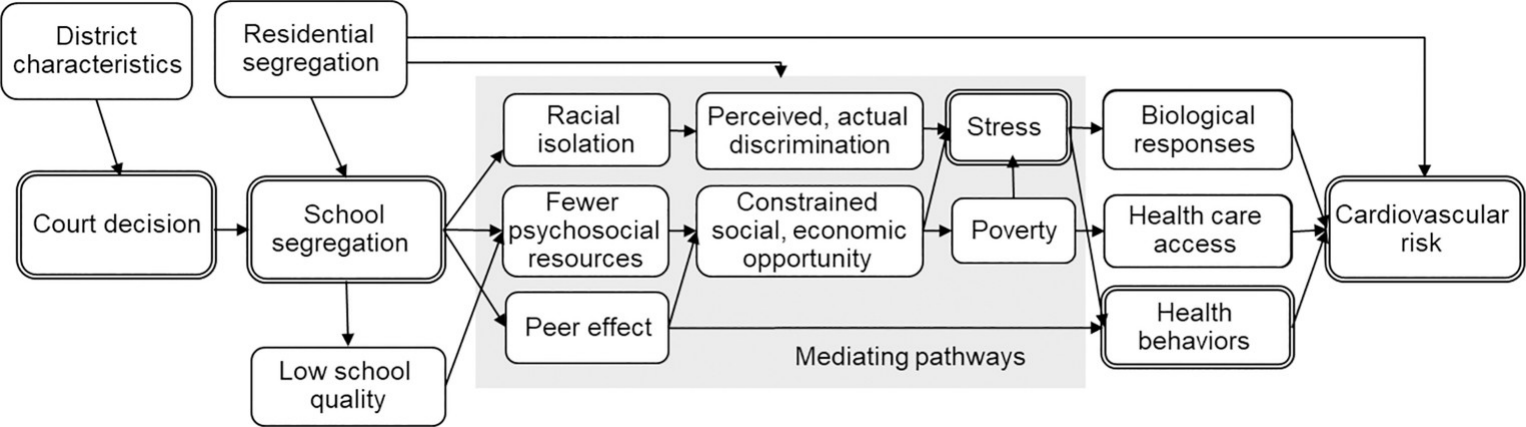
Conceptual model illustrating the pathways linking school racial segregation with cardiovascular risk. Boxes with double borders represent the exposure, instrument, and outcomes of interest.

School racial segregation may be linked to CVD and its risk factors through multiple mechanisms (see causal diagram/conceptual model in Fig 1). First, school segregation may lead to reduced school quality (such as high teacher turnover or limited material resources) [28,29], in part through reduced federal, state, and local funding for schools serving Black students (particularly in predominantly non-White school districts) [30,31]. This may constrain social and economic opportunities and result in reduced educational attainment and income later in life [19,32–34]. This, in turn, may increase CVD risk via the chronic stress of poverty [35], decreased access to healthcare and other health-promoting resources [36,37], and adoption of unhealthy coping behaviors (e.g., smoking and drinking) [38–41]. School segregation may also affect peer networks and reshape behavioral social norms, given collective exposure to segregation-induced adversity. Through peer influence, this may encourage early initiation of smoking, drinking, and reduced physical activity [42–44]. School segregation may also increase racial discrimination against Black students, including actual and perceived discrimination in segregated schools [45–47] or discrimination in racially integrated schools due to racist hostility or implicit bias from White teachers and peers [48,49]. High levels of stress associated with racism [50] may lead to reduced “mental bandwidth” and a greater psychological reliance on unhealthy coping strategies, which may lead to poorer decision-making around unhealthy behaviors [42,51,52]. Heightened stress may also directly promote the “biological embedding” of disease—including CVD—through pathogenic metabolic and inflammatory pathways [53,54]. Prior work suggests that chronic stress related to racism contributes to hypertension and other health problems among Black adults [55–57]. Empirical evidence is needed to inform these theoretical models and provide evidence for interventions and policies.

This study examined the association of school racial segregation among Black children with CVD risk later in life, leveraging a natural experiment induced by the quasi-random (i.e., arbitrary) timing of local court decisions that released school districts from court-ordered desegregation [15–18]. We applied an instrumental variables (IV) analysis to a large national cohort to provide rigorous evidence on whether recent increases in school racial segregation were associated with Black adults’ cardiovascular health.

## Methods

This study involved combining school district childhood exposure data (1991 to 2013) with adulthood health data and applying an instrumental variables method (described below). All analyses were prespecified (S1 Prospective Plan). This study is reported as per the Strengthening the Reporting of Observational Studies in Epidemiology guideline (S1 STROBE Checklist).

### Data

We used de-identified data from the Panel Study of Income Dynamics (PSID), a longitudinal survey of American families that interviewed a nationally representative sample of approximately 4,800 US households in 1968 and followed respondents and their descendants annually through 1997 and biennially thereafter [58]. To connect PSID to school segregation data, we first generated a crosswalk that mapped census blocks onto school districts, conducting a spatial merge using ArcGIS software. Using this crosswalk, we then matched each PSID participant’s residential census block at each survey wave during 1991 to 2013 with school district exposure data, compiled by Reardon and colleagues [59] and ProPublica [60]. As of 2016, about 90% of US children attend public schools: 75% attend their assigned schools, and 15% attend other schools within their assigned district. These values are similar to the range during our study period [61]. Thus, we expect fewer than 10% of children in home schooling or private schools to have been misclassified in this approach using district-level exposures [61].

### Sample selection

We included all individuals who (1) self-identified as Black; (2) contributed at least 1 wave of data during ages 5 to 17 (schooling years); and (3) reported at least 1 adult CVD-related health outcome between 1992 and 2017, totaling 2,137 respondents. Because respondents who had never lived under court-ordered desegregation may differ in important ways, we restricted the sample to respondents who had ever resided in a school district that was under court order as of 1991 during ages 5 to 17, yielding 1,059 individuals from 112 school districts (see S1 Text for additional information about sample selection). Since we are looking at the particular sample who ever received schooling in districts that were under a desegregation order in 1991, our analysis is not intended to be nationally representative. Among 1,059 observations, only 1 case had a missing value for a key covariate, childhood household income, which has been otherwise imputed by PSID for other participants who do not report that variable. Thus, we performed a complete case analysis since such low levels of missingness are not thought to result in substantial bias [62,63]. Finally, we excluded respondents who were the only person in the sample with a given birth year or living in a given state (*n* = 5) to avoid small cell sizes and unstable estimates. The final sample included 1,053 individuals contributing 4,723 person-year observations (mean 4.5 observations per person, median 4, range 1 to 16) (S1 Fig). The number of observations for each model varied since not every outcome measure was captured for all participants in all waves (see Table 1).

**Table 1:** Sample characteristics, Panel Study of Income Dynamics, 1991–2017.

Characteristic	Mean/%	SD	
**Individual sociodemographics** ^**a**^			
Female (%)	60.11		
Birth year	1982	5.46	
Household income per capita (USD)	10,375	9,711	
Parent marital status (%)			
Married	43.53		
Single	26.32		
Separated/divorced/widowed	30.15		
**Individual health outcomes**			No. obs
*Continuous outcomes*			
Psychological distress	4.17	4.21	4,041
Number of cigarettes	2.14	5.10	4,572
Hours of vigorous phys. activity per week	2.16	3.18	4,101
Hours of light phys. activity per week	2.83	3.94	3,714
Body mass index (kg/m^2^)	28.97	6.67	4,547
*Binary outcomes (%)*			
Good health	85.82		4,698
Smoking	23.69		4,576
Alcohol use	62.04		4,586
Binge drinking	19.44		4,522
Heart disease	1.24		4,589
Hypertension	14.78		4,587
Diabetes	2.79		4,587
**Instrument and exposure**			
Proportion of observed schooling in released districts	0.12	0.24	
Average Black–White dissimilarity index	0.45	0.21	
**Baseline school district covariates** ^**b**^			
Total number of students enrolled	101,536	156,102	
Black students (%)	49.61		
White students (%)	30.24		
Hispanic students (%)	16.58		
Receiving free/reduced-price lunch (%)	59.12		
Residential segregation	0.58	0.21	

Note: Sample includes 1,053 Black people (contributing 4,723 person-year observations) who had ever resided as a child in a school district that was under a court desegregation order as of 1991.

^a^Household income and parental marital status reflect the value during the first schooling year observed for each individual.

^b^Denoted measures capture 1991 characteristics of the school district in which each respondent resided at the earliest age observed.

SD, standard deviation; USD, US dollars.

### Variables

#### Exposure

School racial segregation was measured using the Black–White dissimilarity index, the most commonly used metric to detect within-district distributional changes in school racial composition [17,24]. It represents the proportion of Black or White students who would need to change schools in order to achieve a uniform distribution of Black and White students across a district (range: 0 to 1) [64]. A dissimilarity index of 0.5, for example, means that a hypothetical Black student received schooling in a district where 50% of Black or White students would need to move to another school to achieve Black–White racial balance across schools. Our main exposure was operationalized as the mean level of school segregation during schooling years (ages 5 to 17) for observed waves.

#### Instrument

For the IV approach (described below), we operationalized the instrumental variable as the proportion of observed schooling years that each child spent in released school districts. We divided the number of observed waves a child spent in school districts that were released from the 1991 desegregation order by the total number of that child’s observed waves during their schooling years.

#### Outcomes

We included cardiovascular risk factors and outcomes observed up to 20 years after the exposure period. Binary outcomes included self-rated health (good, very good, or excellent versus poor or fair), current smoking status, whether the respondent ever drank alcohol, and binge drinking status (3+ drinks per day). While the latter does not perfectly accord with the official definition of binge drinking (4 or more drinks per day for women and 5 or more for men) [65], PSID does not include a consistently worded question for alcohol consumption across survey waves. We also included self-reported diagnoses of heart disease (coronary heart disease, angina, or congestive heart failure), hypertension, and diabetes (type unspecified). Continuous outcomes included stress, measured with the Kessler-6 Psychological Distress Scale (range 0 to 24) [66], number of cigarettes per day (coded as 0 for nonsmokers), hours of light physical activity per week, hours of vigorous physical activity per week, and body mass index (BMI).

#### Covariates

We selected individual and school district covariates that represent potential confounders, impacting both exposure and outcomes [67–69].

Individual-level covariates included sex (binary), baseline household inflation-adjusted income per capita, baseline parental marital status, and indicator variables for birth year to account for secular trends, as well as state indicator variables (i.e., fixed effects) corresponding to the first observed state of residence to account for confounding due to measured and unmeasured time-invariant state-level characteristics.

We also adjusted for 1991 school district covariates derived from the Common Core of Data from the National Center for Education Statistics [70], including total enrolled students; percent of students who were White, Black, and Hispanic; percent eligible for free or reduced-price lunch; and 1990 residential racial segregation, measured by the Black–White dissimilarity index (computed using tracts nested within districts) and generated from the decennial census. For respondents who changed school districts during their schooling years, we chose the first observed school district for covariate adjustment.

### Analysis

We first tabulated participant characteristics. Next, we estimated effects using 2 types of models in the sample pooled across waves: (1) a standard linear model estimated by ordinary least squares (OLS), which may suffer from confounding of the relationship between school segregation and CVD; and (2) IV analysis, intended to address this confounding.

In OLS analyses, we regressed each outcome on school racial segregation. We adjusted for the covariates listed above. We employed Huber–White robust standard errors clustered at the individual level and district level to account for the potential correlation of observations over time and within school districts [71]. We carried out linear regressions for both binary and continuous outcomes. Results for binary outcomes should therefore be interpreted as a risk difference, i.e., a change in percentage point risk of the outcome. OLS estimates, however, may suffer from bias if there is confounding by factors that are not included as covariates in standard linear models. These might include unobserved individual (e.g., genetic endowment), family (e.g., parental occupation), or area-level (e.g., neighborhood) characteristics.

We therefore next performed IV analysis, an established quasi-experimental approach that reduces the threat of unmeasured confounding [67,72]. IV analysis is particularly useful when the predictor cannot be easily randomized [67], as in the case of school segregation. It is designed to address situations in which the relationship between the predictor and outcome may be confounded by unobserved factors (S2 Fig); in this case, school segregation and CVD risk may both be determined by other characteristics like family socioeconomic status or residential segregation. IV analysis relies on the existence of a quasi-randomly determined exposure—known as the “instrument”—that impacts the predictor but does not suffer from the same unobserved confounding. The quasi-random perturbation in the predictor (i.e., school segregation) caused by the instrument (i.e., timing of local court decisions) is then used to infer the effects of the predictor on the outcome. In practice, this involves a 2-stage regression, with multivariable models adjusting for the covariates above and standard errors clustered by individual and school district.

We also ran models in which we regressed each outcome directly on the instrument to estimate the association of desegregation order releases with health outcomes (known as the “reduced form” of the IV analysis) [73]. Reduced form models ascertain the plausibility of any observed IV effects; they are often of interest to practitioners since they estimate the effects of the social policy itself on CVD, while the IV models leverage these policy changes to estimate the effects of school segregation on CVD. All analyses were performed using Stata 14 (College Station, Texas).

#### IV assumptions and validation

IV analysis rests on several assumptions, including (1) the instrument must be a cause of the exposure, which in this case is school segregation (the relevance assumption); (2) the instrument only affects the outcome through the exposure (the exclusion restriction); and (3) the instrument does not share unmeasured common causes with the outcome (the exchangeability assumption) [69].

We formally tested the first assumption (relevance) by regressing the exposure on the instrument and other covariates, which is known as the “first stage” of the IV analysis [72]. F-statistics from the first stage of IV models were well above the standard cutoff of 10 (e.g., 41.09 for self-rated health) (Table A in S1 Text). This indicates that the proportion of years spent in a released district is a strong instrument for school racial segregation and supports the first assumption of the IV approach.

The second assumption (exclusion restriction) would be violated if the court decisions resulted in a change in CVD risk through some pathway other than altering school segregation. For example, this assumption would be violated if neighborhood characteristics changed in a given district in response to a local court decision and also affected CVD. While this assumption is not directly testable, we believe it unlikely that families relocated following a court order in their district [74]. Empirical evidence also supports few changes in residential segregation levels in released districts [75]. We further describe below several sensitivity tests to explore this issue.

Finally, the third assumption (exchangeability) would be violated if there were unmeasured common causes of both the instrument and outcomes. While there are observed differences in the characteristics of school districts that were eventually released from court desegregation orders compared to districts that were not dismissed (e.g., racial composition, enrollment size) [17,24], prior work has demonstrated that the *timing* of court decisions was subject to “an element of randomness” due to a combination of forces unrelated to individual students’ CVD risk (e.g., unequal caseloads across district courts, varying judicial processes, appeals on decisions). This results in arbitrary (i.e., quasi-random) timing of districts’ releases [17,24,26]. If this assumption is met, releases resulted in quasi-random exposure of children to resegregation in affected districts.

While we cannot formally test the second and the third assumptions, we implemented 2 falsification tests to provide additional evidence of the validity of the instrument [67,73,76]. First, for the exclusion restriction assumption, we ran similar IV analyses among Black adults living in the same districts who were not of school age at the time releases occurred (i.e., an untreated group). Null IV estimates for the untreated group would support our assumption that court order decisions only affected CVD outcomes through school segregation rather than other channels, such as neighborhood characteristics. Second, for the exchangeability assumption, we ran the same IV model using height as a placebo outcome that may be affected by a potential confounder but unlikely to be affected by school segregation [77,78]. The results of these tests validated the model assumptions; additional details of these results and other aspects of the IV analysis, including equations, are provided in Tables B, C, and D in S1 Text.

#### Robustness checks

We carried out 3 types of sensitivity analyses to support the robustness of findings in the main models: (1) running probit models for binary outcomes to ensure estimators from the main linear probability models are reliable; (2) limiting the sample to participants with more than 7 years of observed schooling years to ensure the exposure values we assigned were not biased for respondents with fewer observations across their childhood; and (3) using the Black isolation index as an alternative operationalization of school segregation (Tables E, F, and G in S1 Text).

#### Multiple hypothesis testing

Given the number of tests we conducted, we calculated adjusted *p*-values using the Romano–Wolf method, which controls for the familywise error rate using a resampling approach [79,80].

### Ethics approval

The present study was approved by the institutional review board of the senior author’s institution (protocol 18–25536).

## Results

### Sample characteristics

Three-fifths of the sample were female, the mean birth year was 1982, and the mean household income per capita was about $10,400 (Table 1). On average, participants spent 12% of their observed schooling years in released districts. The average level of school segregation (i.e., Black–White dissimilarity index) that participants experienced during their schooling years was 0.45 (SD 0.21). This estimate represents a moderate level of segregation, with prior literature using a threshold above 0.6 to indicate high segregation [81,82]. The mean age at outcome measurement was 28 years (range 18 to 44).

### Association of school segregation with CVD risk (standard linear models)

**Fig 2:**
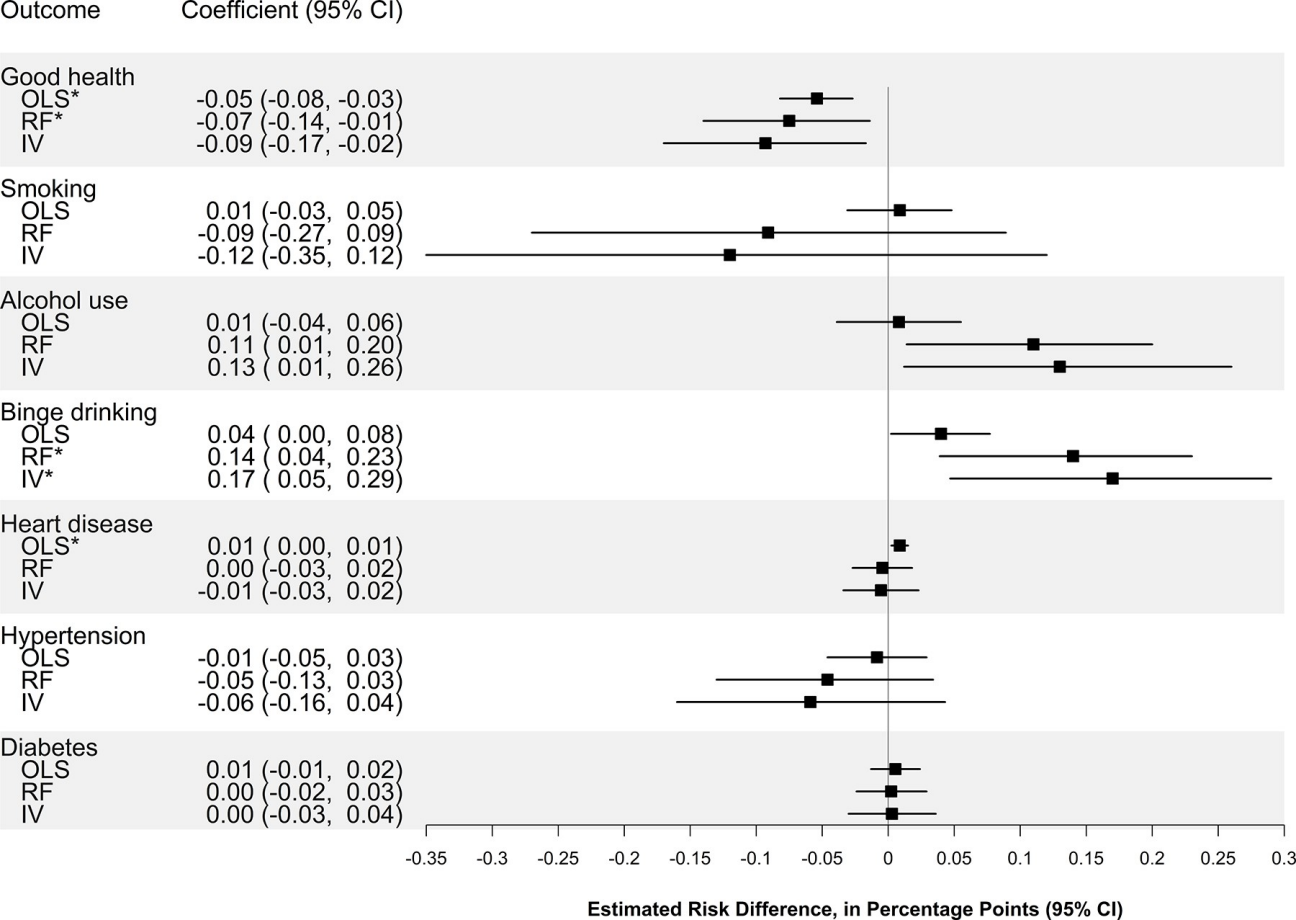
Association of school racial segregation with adult cardiovascular disease risk (binary outcomes). The squares represent the point estimate of the change in outcome per SD of the dissimilarity index, and the bars represent the 95% CIs. Estimates are derived from linear models adjusting for individual and school district characteristics as well as birth year and state fixed effects, with standard errors clustered at the individual and school district levels among 1,503 Black people from the PSID who had ever resided as a child in a school district that was under a court desegregation order (as of 1991) and had health outcomes observed during adulthood. * Indicates *p*-value <0.05 after adjustment for multiple hypothesis testing. IV, instrumental variables; OLS, ordinary least squares; PSID, Panel Study of Income Dynamics; RF, reduced form.

**Fig 3:**
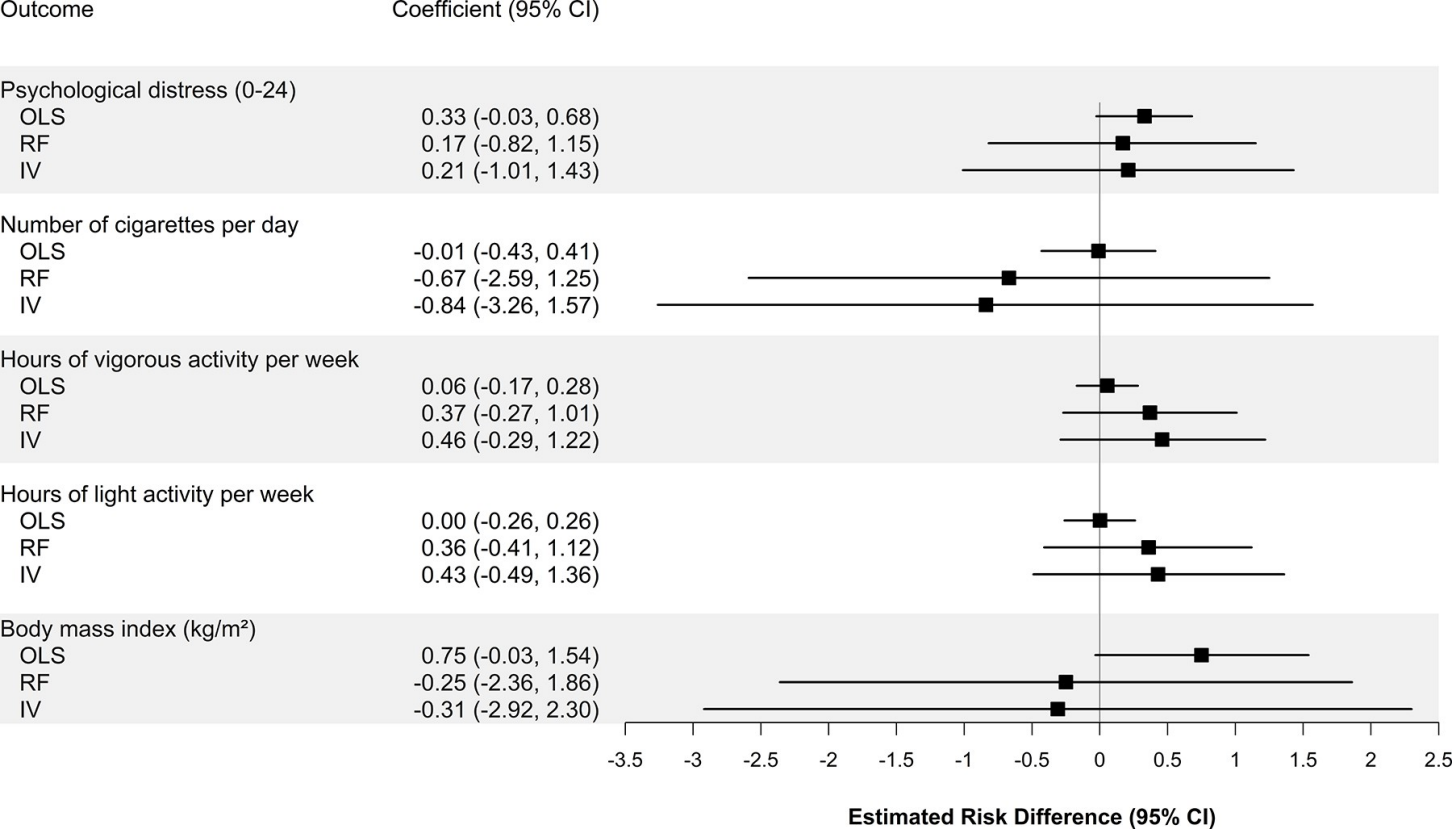
Association of school racial segregation with adult cardiovascular disease risk (continuous outcomes). The squares represent the point estimate of the change in outcome per SD of the dissimilarity index, and the bars represent the 95% CIs. Estimates are derived from linear models adjusting for individual and school district characteristics as well as birth year and state fixed effects, with standard errors clustered at the individual and school district levels among 1,503 Black people from the PSID who had ever resided as a child in a school district that was under a court desegregation order (as of 1991) and had health outcomes observed during adulthood. IV, instrumental variables; OLS, ordinary least squares; PSID, Panel Study of Income Dynamics; RF, reduced form.

Standard linear models demonstrated that school segregation was associated with a lower probability of good, very good, or excellent health (−5 percentage points per standard deviation in the dissimilarity index; 95% CI: −8, −3; *p* < 0.001) and a higher probability of binge drinking (4 percentage points; 95% CI: 0.2, 8; *p* = 0.04) and heart disease (0.9 percentage point; 95% CI: 0.2, 1.5; *p* = 0.007) (Fig 2). We were unable to reject the null hypothesis of no association for other outcomes (Figs 2 and 3).

### Effect of school segregation on CVD risk (IV models)

IV analyses confirmed the results from standard linear models, indicating that increased school racial segregation was associated with a lower probability of good to excellent health (−9 percentage points, 95% CI: −17, −2, *p* = 0.02) and a higher probability of binge drinking (17 percentage points, 95% CI: 5, 29, *p* = 0.008). These analyses also showed that school segregation was associated with a higher probability of alcohol use (13 percentage points; 95% CI: 1, 26; *p* = 0.03) (Fig 2). We were unable to reject the null hypothesis of no association for other outcomes (Figs 2 and 3).

### Association of release from court-ordered desegregation with CVD risk (reduced form models)

Reduced form models showed that more time spent in districts that had been released from court-ordered desegregation was associated with a lower probability of good to excellent health (−7 percentage points; 95% CI: −14, −1; *p* = 0.02) and a higher probability of alcohol use (11 percentage points; 95% CI: 1, 20; *p* = 0.03) and binge drinking (14 percentage points; 95% CI: 4, 23; *p* = 0.006) (Fig 2).

### Multiple hypothesis testing

After calculating adjusted *p*-values using the Romano–Wolf method, results remained statistically significant at a *p*-value of 0.05 for self-rated health and heart disease in standard linear models, self-rated health and binge drinking in reduced form models, and binge drinking in IV models (Table H in S1 Text).

## Discussion

This study leveraged a natural experiment that resulted in quasi-random variation in respondents’ exposure to school racial segregation, providing rigorous evidence on the association of a recent increase in school segregation with CVD and its risk factors. Standard linear models and IV models (which more rigorously adjusted for confounding) demonstrated that school segregation was associated with a lower probability of good self-rated health and a higher probability of binge drinking. Specifically, in IV models, a 1 standard deviation increase in the segregation index (about 0.2) was associated with a 9 percentage point decrease in the probability of having good health (about an 11% relative change) and a 17 percentage point increase in the probability of binge drinking (about an 87% relative change). These magnitudes are comparable to effect estimates of other social exposures on these types of outcomes, such as a 1-year increase in educational attainment [83] or moving out of high-poverty neighborhoods [84]. Although the magnitude of these effect sizes may be modest at the individual level, it is substantial at a population level [85].

When we implemented a procedure to account for multiple hypothesis testing, the association between school segregation and binge drinking in IV models remained statistically significant, but we were unable to reject the null hypothesis of no association for other outcomes.

Our finding that school segregation is associated with unhealthy drinking behaviors adds evidence to the literature documenting associations between early-life and neighborhood-level disadvantage with Black adults’ alcohol use [38,86–88]. This is also consistent with recent findings that school resegregation leads to the development of behavioral issues and increased unhealthy drinking behaviors during childhood [89]. There are multiple mechanistic pathways that potentially link school segregation and adult drinking behaviors. Attending racially segregated schools, which are often underfunded [30,31] and tend to have a high concentration of students whose families do not have the material resources or political capital to overcome this barrier [28], may result in a lower quality education. This can adversely impact educational attainment, the concentration of resources within people’s social networks, labor market opportunities, and future earnings [29,33,34,90,91]. Moreover, students in segregated schools are exposed to discrimination, including perceived discrimination (e.g., due to a sense of exclusion) and actual discrimination (e.g., due to harsher treatment and discipline of students at racially segregated schools, part of the “school-to-prison pipeline”) [45–47]. Accumulated stress associated with socioeconomic constraints and experiences of racism may, in turn, lead to poor health among Black adults and heavy drinking as a coping strategy [38,92]. Early drinking engagement induced by school resegregation [89] may set children on a trajectory leading to heavy drinking in adulthood [93].

Standard linear models estimated by OLS suggested that school segregation was associated with an increased risk of self-rated health and heart disease, although these were not replicated by IV analyses after adjusting for multiple hypothesis testing. Several scenarios could explain these contrasting findings. First, the standard linear model results may reflect unmeasured confounding, and the IV results may reflect the true absence of a relationship between school segregation and these outcomes. For example, Black children who attended segregated schools may have greater exposure to built environments (e.g., fast food restaurants) that increase CVD risks [94], or children with few resources, especially those whose parents have CVD and who may have passed on that risk to their children through genetic or epigenetic mechanisms [95], may be sorted into segregated schools. The effects observed in prior research linking school segregation with child health [89] may wane as children age. Second, there may in fact be an association, but this may not be captured because the IV estimates are imprecise [96]: Confidence intervals from IV models were wide and overlapped with point estimates from standard linear models. The strength of IV analysis is that it reduces biases that are common in standard linear models due to unmeasured confounding, but it does so at the expense of statistical precision due to 2-stage estimation [97]. Future studies should therefore replicate these analyses in larger samples. Third, standard linear and IV models may not be measuring the impact of school segregation caused by the same forces. Standard linear models relied on variation in school segregation by all causes, conditional on confounders. IV analyses, in contrast, relied on variation in school segregation due to court orders. That is, IV models captured a “local average treatment effect,” or the effect on “compliers” whose exposure to school segregation was influenced by court orders [98,99]. More research is needed to assess whether school segregation resulting from other policies (e.g., housing programs) produces similar estimates.

The current study adds timely evidence on the health effects of race-based inequities in the education system, given concern over resegregation in recent decades. Since the Supreme Court’s 1991 ruling in Board of Education of Oklahoma City v. Dowell and subsequent cases, states and school districts have largely replaced explicitly segregationist policies with a de facto regime of school segregation that relies on residential segregation [14], investing in education unequally by geographic areas delineated by race. This residential segregation—itself due to historical, explicitly racist housing policy and current housing practices that disproportionately impoverish people of color—allows educational segregation to be legal again, without having to enforce it via explicitly racist schooling policies. This indicates that educational equity was not fully achieved under Brown and casts doubt on the effectiveness of past schooling policies for improving educational equity. Nevertheless, previous studies found that school desegregation during the 1960s to 1970s improved Black American’s well-being in many aspects, such as educational and occupational attainment and overall health [19,20]. Thus, the current finding that the policy change allowing resegregation was associated with greater CVD risk among Black adults is broadly consistent with the early evidence.

This study contributes to policy discussions about the importance of schooling policies and the need for new efforts to promote educational and health equity, such as reforming school funding formulas to increase government funding and resources for disinvested schools in segregated districts [100]. At the federal level, legislation like the Strength in Diversity Act would use federal funds to support planning and implementing strategies to improve diversity and reduce or eliminate racial or socioeconomic isolation in publicly funded schools [101]. This paper also sets the stage for future studies to investigate the pathways through which school segregation may influence CVD to inform downstream interventions in the community or school setting.

This study has several limitations. First, it did not account for sub-district characteristics (e.g., schools or classrooms). This was in part because the goal was to leverage quasi-random variation in school resegregation caused by changes in court order status at the district level, a theoretically meaningful and policy-relevant construct [17,64,102]. Moreover, PSID does not have data on the specific schools attended by children in the sample. Second, we did not examine cardiovascular outcomes like myocardial infarction and stroke because they were rare in this relatively young sample (mean age 28) [103]. Future studies should examine longer-term outcomes as this cohort ages. Third, IV estimates would be biased if a release from a desegregation order influenced adult CVD risks via mechanisms other than school segregation or if there were unmeasured common causes of court decisions and health outcomes. Our analyses suggest that the IV assumptions were unlikely to be meaningfully violated, but attempts to eliminate desegregation may be intimately linked to a system of parallel laws, policies, and practices that may increase racial CVD disparities in other ways. Additionally, we were not able to include district fixed effects in the models due to the small number of children per district and to avoid over-parameterization. Furthermore, we employed linear models for binary outcomes, which may lead to biased estimates in standard errors [104]. However, our probit models in Table E in S1 Text were similar to the main results from linear models, suggesting that our results are not substantially biased. We note, though, that the sample and model specification were different in probit models, as described in S1 Text. Also, we coded nonsmokers as 0 for the outcome “number of cigarettes” since CVD risk increases as smoking intensity increases [105]. This might be modeled differently. Lastly, self-reported outcomes and covariates may be subject to measurement error or standard reporting biases, although we have no reason to think these biases would be differential by treatment or levels of the IV [106,107].

In conclusion, this study provides rigorous estimates of the association of school segregation—a pervasive and persistent exposure for many Black youths—with long-term CVD and its risk factors. It provides evidence by using a natural experiment and an IV approach to improve upon results from more traditional correlational methods. This study thereby advances the literature on racial inequities in CVD and contributes to a broader and much-needed body of work on the health impacts of school segregation as a form of structural racism in the US.

## Supporting information

S1 STROBE Checklist Checklist of items that should be included in reports of cross-sectional studies. (DOCX)

S1 Prospective Plan This document presents our prospective analytic plan as described in the NIH grant proposal that funded this work (# R01-HL151638). (PDF)

S1 Fig Flowchart of sample selection. (PDF)

S2 Fig Instrumental variables design. (PDF)

S1 Text Supplemental methods. Table A. First-stage coefficients and F-statistics. Table B. Balance test: Comparison of observed characteristics among PSID respondents by instrumental variable. Table C. Falsification test: Association of school racial segregation with cardiovascular risk in PSID sample unexposed to resegregation. Table D. Falsification test: Association of school racial segregation with placebo outcome in the PSID court order sample. Table E. Association of school racial segregation with adult cardiovascular risk, using probit regression for binary outcomes. Table F. Association of school racial segregation with adult cardiovascular risk, in the sample with 7 or more observed schooling years. Table G. Association of school racial segregation (operationalized as the Black isolation index) with adult cardiovascular risk. Table H. Adjusted *p*-value for multiple hypothesis testing.
(PDF)

## Abbreviations

CVD: cardiovascular disease
IV: instrumental variables
OLS: ordinary least squares
PSID: Panel Study of Income Dynamics
