# Supplementary material for: School racial segregation and long-term cardiovascular health among Black adults in the US: A quasi-experimental study
Source: PLoS Med. 2022 Jun 21;19(6):e1004031. doi: 10.1371/journal.pmed.1004031 (PMC9258802; doi:10.1371/journal.pmed.1004031)
Supplement: S1 Prospective Plan — (PDF) [file pmed.1004031.s002.pdf]

S1 Prospective Plan

This document presents our prospective analytic plan as described in the NIH grant proposal that funded this work (# R01-HL151638). There are a few differences between the current manuscript and the original proposal. One is that we now use district-level residential segregation as a covariate, instead of census-tract level, because it is at the same geographic level as our exposure of interest (i.e., school segregation). At the time this proposal was written, we did not have access to district-level residential segregation data. We also did not implement logistic models as there is no standard Stata package for IV logistic analyses. Additionally, the current manuscript also includes additional analyses suggested by reviewers, such as implementing probit analyses to accommodate binary outcomes. Finally, the sample size in our final manuscript for whom we had data on both the outcomes and exposure was smaller than the sample size that we originally anticipated based on our preliminary analyses, particularly after restricting the sample to Black individuals.

<u>Approach</u>

Data Sources

We will link exposure data on school segregation and relevant court decisions from the Stanford Education Data Archive (SEDA) with outcome data on cardiovascular disease (CVD) risk factors from a large diverse U.S. cohort study, the Panel Study of Income Dynamics (PSID). PSID includes data on census block of childhood residence, which we will use to link information on the school district to which that child would have been assigned.

Analytic Plan

Exposure. The primary exposure is a measure of racial segregation while children were in school. For PSID, most children have multiple measures of the segregation index when they were in school; these will be averaged to obtain a mean level of exposure to school segregation during childhood. In an alternative specification, we will also consider the maximum value of school segregation to which each child was exposed.

Outcomes. PSID has queried adult participants since 1999 on self-reported smoking (including current smoking status and number of cigarettes per day), diabetes, and hypertension. It has also included psychological distress since 2001, in the form of the Kessler-6 scale.

Covariates. Our models will adjust for potential confounders at the individual level and family level. We will also adjust for residential segregation at the census tract level, as well as state fixed effects (i.e., indicator variables) which account for any unobserved

time-invariant state-level confounders. Finally, we will adjust for year to account for secular trends.

Statistical Analysis: Standard. We will first carry out standard models, regressing each outcome on the segregation index, while adjusting for covariates. We will employ ordinary least squares (OLS) models for continuous outcomes and logistic models for binary outcomes. The inclusion of state fixed effects means that we only compare school districts to others within the same state rather than across states.

Statistical Analysis: Instrumental Variables (IV). We will next carry out IV analysis, a quasi-experimental method increasingly used for the analysis of observational data in epidemiologic research. Here we take advantage of temporal and geographic quasi-random variation in court decisions (the instrument) that resulted in resegregation of specific school districts. For the IV analysis, we will restrict the sample to those school districts that were ever under court-ordered desegregation, as other school districts may not be comparable.

For PSID, because children are potentially observed multiple times during childhood, the court decisions will serve as an instrument for the *mean* level of school segregation during childhood. (In an alternative specification, we will also consider the maximum value of school segregation to which each child was exposed.)

Power Analysis. For PSID, our preliminary linkages have demonstrated at least 8,000 students for whom we have data on residential addresses in childhood and CVD outcomes during adulthood. Of these, about 4,000 lived in roughly 380 school districts that were subject to court-ordered segregation, and about half of these lived in school districts that were subsequently released and resegregated. Power analyses indicate that the detectable Cohen's effect size index (d) for this analysis is 0.0005, below the 0.02 that is considered small. Even if we assume that observations within the 380 school districts are perfectly correlated, which is unlikely, the detectable Cohen's d would be 0.02.
