# Supplementary figures and images for: School racial segregation and long-term cardiovascular health among Black adults in the US: A quasi-experimental study
Source: PLoS Med. 2022 Jun 21;19(6):e1004031. doi: 10.1371/journal.pmed.1004031 (PMC9258802; doi:10.1371/journal.pmed.1004031)

**S1 Figure.** Flowchart of sample selection

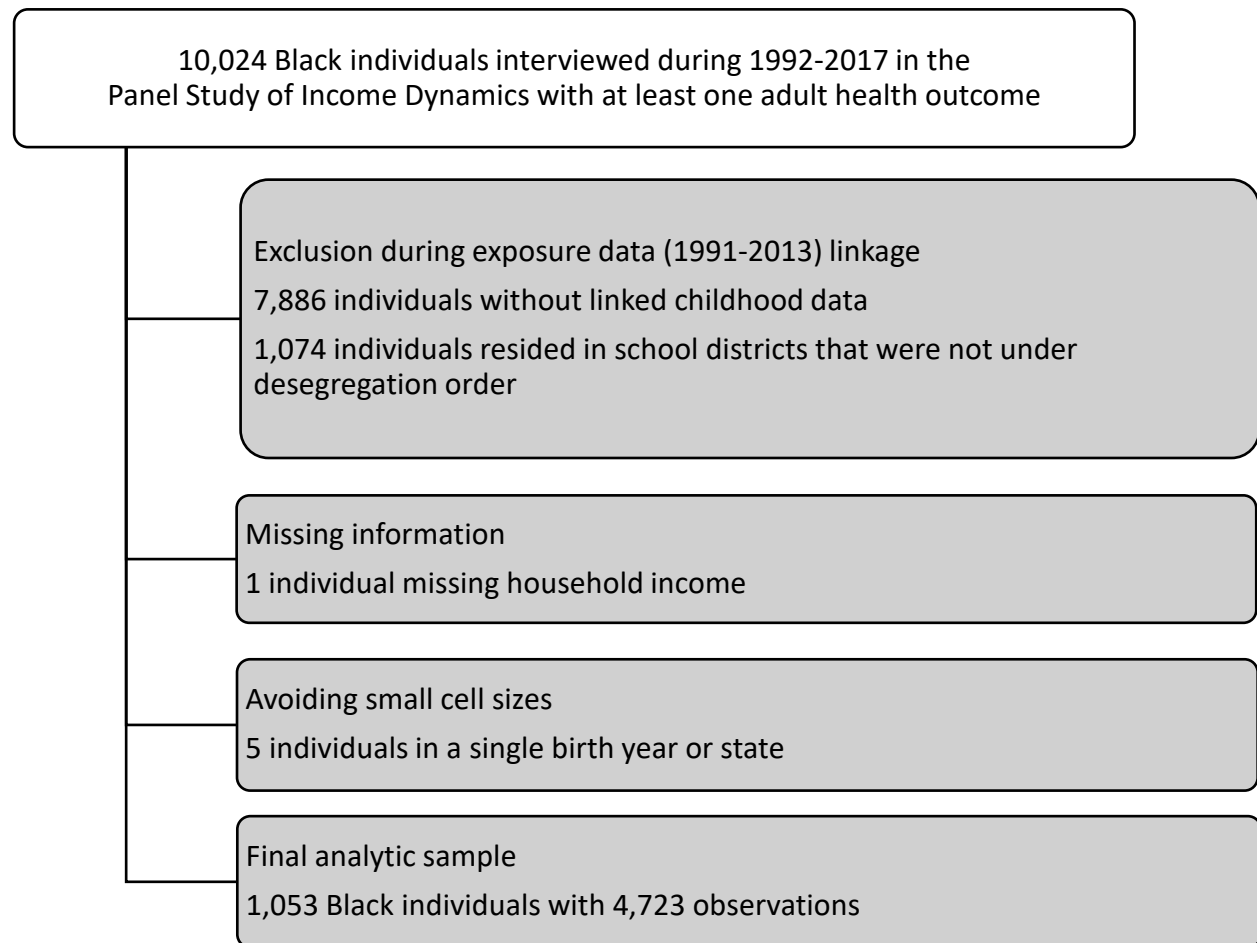

Supplement: S1 Fig — (PDF) [file pmed.1004031.s003.pdf]
