# Supplementary material for: School racial segregation and long-term cardiovascular health among Black adults in the US: A quasi-experimental study
Source: PLoS Med. 2022 Jun 21;19(6):e1004031. doi: 10.1371/journal.pmed.1004031 (PMC9258802; doi:10.1371/journal.pmed.1004031)

S2 Figure. Instrumental variables design. Directed acyclic graph (i.e., causal diagram) illustrating the relationship between an instrumental variable Z, an endogenous predictor or treatment X, unmeasured confounders U, and an outcome Y. This method rests on several assumptions, including the assumption that there does not exist a separate causal pathway W linking the instrument and outcome. *CVD*, cardiovascular disease; *SES*, socioeconomic status.

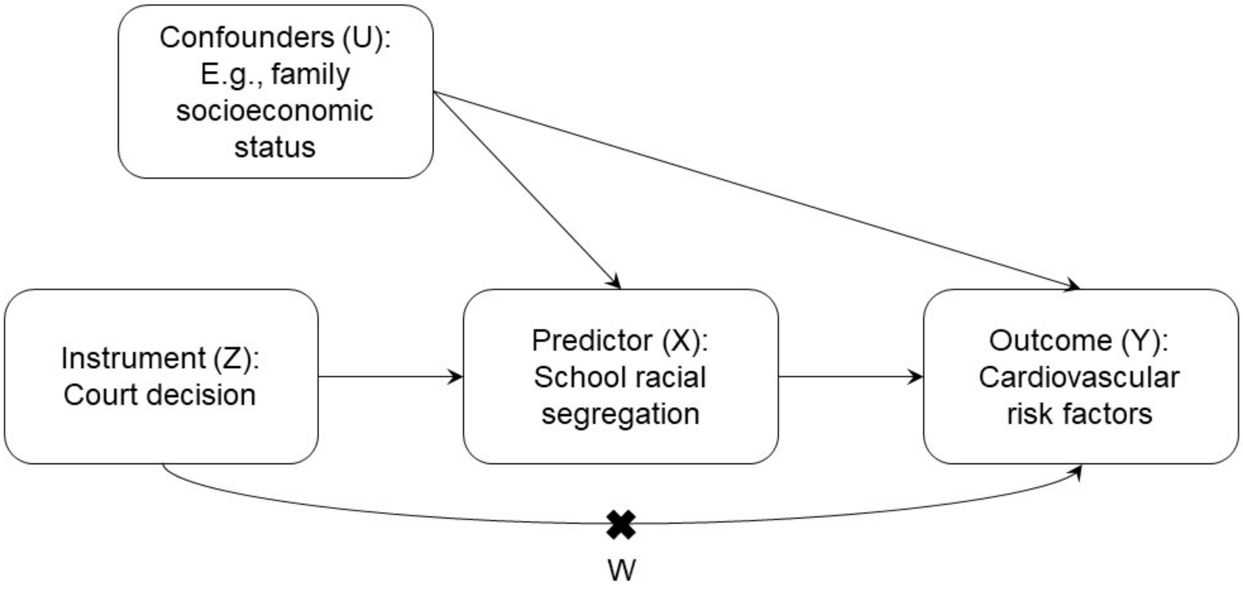

Supplement: S2 Fig — (PDF) [file pmed.1004031.s004.pdf]
