# Supplementary material for: School racial segregation and long-term cardiovascular health among Black adults in the US: A quasi-experimental study
Source: PLoS Med. 2022 Jun 21;19(6):e1004031. doi: 10.1371/journal.pmed.1004031 (PMC9258802; doi:10.1371/journal.pmed.1004031)
Supplement: S1 Text — Table A. First-stage coefficients and F-statistics. Table B. Balance test: Comparison of observed characteristics among PSID respondents by instrumental variable. Table C. Falsification test: Association of school racial segregation with cardiovascular risk in PSID sample unexposed to resegregation. Table D. Falsification test: Association of school racial segregation with placebo outcome in the PSID court order sample. Table E. Association of school racial segregation with adult cardiovascular risk, using probit regression for binary outcomes. Table F. Association of school racial segregation with adult cardiovascular risk, in the sample with 7 or more observed schooling years. Table G. Association of school racial segregation (operationalized as the Black isolation index) with adult cardiovascular risk. Table H. Adjusted p-value for multiple hypothesis testing. (PDF) [file pmed.1004031.s005.pdf]

S1 Text. Supplemental methods.

In this online appendix, we discuss the results of sensitivity analyses and provide additional details about our methods. Sections include:

1. Details about sample restrictions
2. Equations and assumptions of the instrumental variables approach
3. Sensitivity analyses, using 1) probit and IV probit; 2) the sample with more than seven years of observed schooling data; and 3) an alternate segregation measure
4. Adjusted P-values for multiple hypothesis testing

Sample restrictions

We restricted the sample to respondents who had ever resided in a school district that was under court order as of 1991 during age 5 to 17, as students who received schooling in districts that were never under court-ordered desegregation may not represent an appropriate control group. Our goal was to compare students from similar school environments, thereby helping to isolate the effects of court order releases. We constructed exposure data using all childhood geographic locations for available surveys, resulting in a range of 1 to 13 observed schooling years. Because 35% of children in the sample changed school districts at least once during their schooling years, the number of school districts observed in the entire sample of Black children and used for constructing the average school racial segregation measure (N=288) was larger than the number of school districts in the sample that were under a court order in 1991 (N=112). Similarly, for respondents who changed school districts during their schooling years, we chose the first observed school district when creating covariates measures. This resulted the number of school districts used for covariate adjustment was larger (N=159) than the number of school districts in the sample that were under a court order in 1991 (N=112). Note that 608 school districts out of 13,042 total nationwide were subject to court-ordered desegregation as of 1991; 358 of them (58.88%) were dismissed from court oversight as of 2013.

Equations and validations of our instrumental variables approach

OLS and IV Equations

Linear models estimated by ordinary least squares (OLS) are represented by equation (1):

$$CVD_{idst} = \gamma_1 SchoolSeg_{idst} + \gamma_2 X_{idst} + \gamma_3 D_{dst} + \theta_t + \lambda_s + \varepsilon_{idst} \quad (1)$$

Here, CVD_{idst} is a health outcome observed during the follow-up for individual i residing in year t in the school district d located in state s, $SchoolSeg_{idst}$ is the individual's average level of school segregation during observed schooling years, X_{idst} is

a vector of individual-level baseline covariates, D_{dst} is a vector of district-level baseline covariates, θ_t and λ_s represents the birth year and state fixed effects, respectively, and ε_{idst} represents robust standard errors clustered at the district and individual levels. We present the results of linear models for both continuous and binary outcomes to allow for comparability in reporting effect estimates as beta coefficients.

The IV models employed in this study can be represented by the following two equations as IV models are in two stages (commonly known as a two-stage least-squares or 2SLS analysis).

$$SchoolSeg_{idst} = \alpha_0 + \alpha_1 CourtOrder_{idst} + \alpha_2 X_{idst} + \alpha_3 D_{dst} + \theta_t + \lambda_s + \varepsilon 1_{idst} \quad (2)$$

$$CVD_{idst} = \beta_0 + \beta_1 \widehat{SchoolSeg}_{idst} + \beta_2 X_{idst} + \beta_3 D_{dst} + \theta_t + \lambda_s + \varepsilon 2_{idst} \quad (3)$$

In the first stage, which is represented in equation (2), the dismissal from the court order is used to predict school segregation. Specifically, the average level of school segregation during the observed schooling years for each individual was modeled as a function of the IV, the proportion of observed schooling years spent in districts that had been released from court-ordered desegregation during 1992-2013. Other predictors in this model include the vector of the individual- (X) and district-level (D) covariates as well as fixed effects for birth year (θ) and state (λ). The predicted school segregation calculated for each respondent from the first stage is then used in equation (3), with each CVD-related health outcome as a dependent variable, as the second stage of the IV analysis. This predicted segregation is not subject to the same confounding as the individual's actual segregation since the desegregation court order determines it. The coefficient of interest is β_1, which represents the effect of the increase in school segregation on the outcome. Robust standard errors ε1 and ε2 were clustered at the district and individual levels.

IV Assumptions

The IV approach requires a rigorous evaluation of whether the identifying assumptions of the instrument are met to provide reassurance about the validity of the IV design. This is necessary to avoid false or misleading findings [1-3]. For example, an IV estimate would falsely attribute some of the adverse effects of other conditions to school-level segregation if the exclusion restriction assumption was violated (i.e., if local court decisions were related to long-term health outcomes through pathways other than school segregation). Bias may also arise if there are unmeasured common causes of the instrument and our health outcomes, a violation of the third exchangeability assumption. We employed three falsification tests to test these assumptions [2].

First, to identify potential factors that could confound the relationship between the instrument (the proportion of observed schooling years that each child spent in released school districts) and the outcomes, we compared mean values of observed characteristics at baseline between (A) the group of individuals who attended schools in districts released from desegregation court orders and (B) those who received schooling

in districts that were not released (Table B). Some differences were expected: prior work demonstrated an association between district characteristics and release status. Specifically, district racial composition has been found to be related to release status in districts outside of the South, while total district enrollment is positively associated with release in Southern districts [4, 5]. While the descriptive results in our sample also showed some notable differences by release status—for example, individuals who received schooling in released districts were more likely to attend districts with smaller total enrollment—most of these differences were no longer evident when we compared the characteristics of students by the proportion of schooling they received in released districts (<50% vs. >=50%). That is, there were very few differences between the baseline characteristics of sample members that had high vs. low exposure to districts released from court orders. Nonetheless, the first balance test did indicate that adjusting for baseline individual and district covariates in IV models could be necessary to eliminate confounding; we thus included the following individual-level variables in IV models: sex, birth year, household income per capita, and parent marital status. We also adjusted for the following baseline school district characteristics: the total number of students enrolled, the proportion of Black, White, and Hispanic students, the proportion of students receiving free/reduced-price lunch, and residential segregation. Additionally, we included state fixed effects to account for potential state-level differences (e.g., policies) which may have influenced local decisions and cardiovascular health. Notably, a previous study found districts' residential segregation level—a key potential long-term contributor to adverse health among the Black population—remained unchanged after districts were dismissed, suggesting that court releases did not alter critical neighborhood characteristics [6].

Second, we evaluated the validity of the exclusion restriction assumption, i.e., that the instrument only affects the outcome through the exposure. To do so, we used a falsification test, since the assumption cannot be empirically tested. Specifically, we investigated whether there were any health effects among Black adults who were aged 18-30 when the court decisions of interest took place. This alternative population ought not to have been affected by the instrument (i.e., court desegregation order releases) but would be affected by potential confounders correlated with those releases (e.g., racially discriminatory sentiment within the district related to court order releases). If court order decisions only affected the outcomes of interest through school segregation, we should expect null IV estimates for such an untreated group. In this falsification test, IV estimates were statistically insignificant for all outcomes of interest, except for hours of light physical activity (Table C). Most estimates were near zero, and all 95% confidence intervals other than light physical activity included a wide range of values, including large negative and large positive values. If the main findings were driven by unmeasured confounding by factors related to district releases that were also related to the health outcomes, we would expect statistically significant effects mirroring those from the primary analysis in this older population who had already completed K-12 education. The null results provide further reassurance that the IV assumptions were

met. The one exception is for physical activity, whose results in the main analysis should thus be interpreted with caution (although our main results for physical activity were null, so our conclusions about this risk factor remain unchanged).

Third, we evaluated the validity of the exchangeability assumption (i.e., whether there might be unmeasured common causes of the instrument and outcomes). To do so, we analyzed an alternative "placebo" outcome—height in adulthood—that ought not to have been affected by perturbations in school segregation induced by our instrument (i.e., court order releases) but would be affected by potential confounders, such as family background [7, 8], which may be correlated with the instrument. Null IV estimates for this kind of alternative outcome would provide further reassurance that there were no common causes of the instrument and the outcomes of interest. Potential family-level risk factors can impact height (e.g., poverty and malnutrition during childhood) prior to or during school segregation exposure, but levels of severe malnutrition should not be related to changes in school racial segregation exposure induced by the exposure, which would not have affected children until they started school at age 5 or later. The result for this analysis was null (Table D), again reducing the likelihood of residual confounders of the IV-outcome relationship.

Sensitivity analyses

Probit and IV probit models

While prior work has shown that the statistical properties of linear regression for binary outcomes are less problematic with large samples like ours [9], there is a concern that estimators from logistic regression might be more appropriate and reliable for binary outcomes than those from linear probability regression, because a predicted probability may fall outside the range 0–1, and because the relationship between probability and the predictor may not be linear. To account for such a concern, we ran probit models for binary outcomes, given that IV probit models are supported by a standard Stata package. There were two main differences in probit model specifications from the linear models: 1) we only applied clustering at the individual-level because the standard probit and IV probit commands do not allow two-way clustering, and 2) half of observations for heart disease outcome were dropped by Stata in the probit model due to collinearity between some variables. Results showed that estimates from probit models did not substantively differ from linear probability models (Table E).

Limiting the sample to those who had childhood observations in more than 7 waves

We did not observe the entirety of schooling years for many respondents in the sample due to school attendance outside of the window 1991-2013, or due to missed survey waves. For example, we included a respondent with only one observation during their schooling years, at age 17; or a respondent with two observation points, surveyed at age 10 and 16. To ensure the exposure values we assigned were not biased for

respondents with fewer observations across their childhoods, we carried out a sensitivity analysis in which we limited the analytic sample to participants who had more than seven years (the median value) of observed schooling. We lost approximately 50% of the sample via this approach. While results were less precisely estimated (due to the smaller sample size) and attenuated, they were in the same direction as the main findings (Table F).

Using the Black isolation index to measure school segregation

We carried out additional analyses in which school racial segregation was operationalized using the Black isolation index, a commonly used measure in the residential segregation and health literature [10-14]. Black isolation index values (range 0-1) represent the probability that a Black student shares a unit (i.e., school) with other Black students; that is, they evaluate the degree to which Black students are separated from non-Black students. If there is little school segregation (i.e., an even distribution of students of different races across schools), this measure will approach the percent Black for the school district as a whole, whereas this measure will approach 1 if there is extensive segregation, as the schools attended by Black students become more and more homogeneous. The caveat for this measure is that it is highly dependent on district racial composition, where the index is mechanically high once a district contains a larger percentage of Black students [15]. Analyses using the isolation index could thus be confounded by changes in district racial composition. Nevertheless, analyses using the Black isolation index as an alternative school segregation measure yielded similar results to those of models using the Black-White dissimilarity index (Table G).

Table A. First-stage coefficients and F-statistics

	Dissimilarity index		Isolation index	
	Estimate	95% CI	Estimate	95% CI
Proportion of schooling in released district	0.80**	[0.55, 1.04]	0.46**	[0.28, 0.65]
F-statistic	41.12		10.98	

* p <0.05; ** p <0.01. Abbreviation*:* CI, Confidence interval.

Note: Estimates are drawn from the first stage of the instrumental variables regression, where school segregation—operationalized using either the dissimilarity index or the isolation index—was regressed on the instrument (the proportion of schooling in released district) among the sample with the self-rated health outcome. F-statistics for models with other outcomes were similar. Models adjusted for 1991 school district characteristics of the first observed school district, household income and parental marital status at the first observed schooling year, sex, birth year fixed effects, and state fixed effects of the first observed school district. We also clustered standard errors at the individual level and district level.

Table B. Balance test: Comparison of observed characteristics among PSID respondents by instrumental variable

Characteristic	Dismissal status					Proportion of observed schooling years in dismissed districts				
	Not dismissed (N=672)		Ever dismissed (N=381)			<50% (N= 188)		>= 50% (N= 193)		
	Mean/ %	(SD)	Mean/ %	(SD)	t / χ[2] test	Mean/ %	(SD)	Mean/ %	(SD)	t / χ[2] test
Individual demographics										
Female (%)	54.5		52.8		-	50.0		55.44		-
Birth year	1983	5.58	1988	4.70	***	1987	4.59	1989	4.48	***
Household income per capita (USD)[a]	10,338	9,788	9,891	8,913	-	10,756	9,432	9,048	8,315	-
Parent marital status[a]										
Married (%)	44.60		39.40		**	45.21		33.68		-
Single (%)	25.40		36.50			34.04		38.86		
Separated/divorced/widowed (%)	30.00		24.10			20.74		27.46		
Baseline school district covariates[b]										
Total number of students enrolled	120,872	188,946	70,649	72,186	***	67,778	81,443	73,446	61,943	-
Proportion Black students (%)	48.10		52.10		**	53.54		52.15		-
Proportion White students (%)	29.80		30.60			26.82		30.60		-
Proportion Hispanic students (%)	18.30		14.30		*	15.38		14.27		-
Proportion free/reduced-price lunch (%)	59.70		56.70		-	58.61		56.67		-
Residential segregation	0.57	0.24	0.60	0.14	-	0.59	0.20	0.60	0.14	-

* p <0.05; ** p <0.01; *** p <0.001. Abbreviations: PSID, Panel Study of Income Dynamics; SD, Standard deviation; USD, US dollars.
Note: Sample includes 1,053 Black people (4,723 observations) who had ever resided as a child in a school district that was under a court desegregation order as of 1991. Among them, 381 individuals (obs=1,268) received schooling in districts that were released from the court order between 1992 and 2013, while 673 individuals (obs=3,461) attended schools in districts under the desegregation court order. Among dismissed group (N=381), 188 individuals received less than 50% of observed schooling years in dismissed districts, whereas 193 individuals received more than 50% of observed schooling years in dismissed districts.
[a]Household income and parental marital status reflect the value during the first schooling year observed for each individual.
[b]Denoted measures capture 1991 characteristics of the school district in which each respondent resided at the earliest age observed.

Table C. Falsification test: Association of school racial segregation with cardiovascular risk in PSID sample unexposed to resegregation

Characteristic	Estimates	95% CI	Obs.
Continuous outcomes			
Psychological distress	-0.12	[-2.13, 1.89]	5,046
Number of cigarettes	-0.13	[-2.25, 2.00]	6,760
Hours of vigorous physical activity per week	-0.33	[-1.05, 0.39]	6,145
Hours of light physical activity per week	-1.18**	[-2.03, -0.33]	5,359
Body mass index	2.06	[-0.99, 5.12]	6,671
Binary outcomes			
Good health	0.00	[-0.13, 0.13]	7,864
Smoking	0.03	[-0.17, 0.22]	6,774
Alcohol use	0.09	[-0.14, 0.32]	6,791
Binge drinking	0.00	[-0.14, 0.15]	6,683
Heart disease	0.01	[-0.5, 0.07]	6,804
Hypertension	-0.02	[-0.19, 0.14]	6,801
Diabetes	0.04	[-0.07, 0.15]	6,800

* $p < 0.05$; ** $p < 0.01$, Abbreviations: PSID, Panel Study of Income Dynamics; IV, Instrumental variables; CI, Confidence interval.

Note: Estimates are derived from IV analyses using an alternative sample in PSID, who ever lived in school districts that were under desegregation order in 1991 while they were ages 18-30 and who had one or more outcomes measured at age 31 and over during 1992-2017. The endogenous exposure variable was school racial segregation, operationalized as the average dissimilarity index across observed schooling years. The instrument was the proportion of schooling in released districts. We adjusted for 1991 (baseline) school district characteristics of the first observed school district, household income and parental marital status at the first observed schooling year, sex, birth year fixed effects, and state fixed effects of the first observed school district. We also clustered standard errors at the individual level and district level.

Table D. Falsification test: Association of school racial segregation with placebo outcome in the PSID court order sample

Outcome	Estimate	95% CI	Obs.
Height	0.01	[-0.026, 0.037]	4,591

* p <0.05; ** p <0.01. Abbreviations: PSID, Panel Study of Income Dynamics; IV, Instrumental variables; CI, Confidence intervals.
Note: Estimate is derived from IV analysis. The endogenous exposure variable was school racial segregation, operationalized as the average dissimilarity index across observed schooling years. The instrument was the proportion of schooling in released districts. We adjusted for 1991 (baseline) school district characteristics of the first observed school district, household income and parental marital status at the first observed schooling year, sex, birth year fixed effects, and state fixed effects of the first observed school district. We also clustered standard errors at the individual level and district level.

Table E. Association of school racial segregation with adult cardiovascular risk, using probit regression for binary outcomes

	Probit		IV Probit		
Outcome	Estimates	95% CI	Estimates	95% CI	N
Good health	-0.25**	[-0.38, -0.12]	-0.50*	[-0.93, -0.07]	4,692
Smoking	0.03	[-0.12, 0.18]	-0.40	[-0.89, 0.09]	4,572
Alcohol use	0.02	[-0.10, 0.14]	0.36	[-0.02, 0.73]	4,565
Binge drinking	0.16**	[0.05, 0.27]	0.61**	[0.25, 0.97]	4,520
Heart disease	0.54**	[0.24, 0.84]	-0.51	[-1.57, 0.56]	2,232
Hypertension	-0.06	[-0.21, 0.10]	-0.44	[-0.95, 0.07]	4,564
Diabetes	0.08	[-0.18, 0.34]	-0.06	[-0.95, 0.82]	3,736

* p <0.05; ** p <0.01. Abbreviations: IV, Instrumental variables; CI, Confidence interval. Note: Estimates are derived from probit regressions for each binary outcome. The endogenous exposure variable was school racial segregation, operationalized as the average Black-White dissimilarity index across observed schooling years. The instrument was the proportion of schooling in released districts. We adjusted for 1991 (baseline) school district characteristics of the first observed school district, household income and parental marital status at the first observed schooling year, sex, birth year fixed effects, and state fixed effects of the first observed school district. Standard errors were clustered at the individual level only.

Table F. Association of school racial segregation with adult cardiovascular risk, in the sample with 7 or more observed schooling years

Outcome	Estimates	95% CI	N
Continuous outcomes			
Psychological distress	-0.18	[-1.58, 1.22]	2,252
Number of cigarettes	-0.74	[-2.64, 1.16]	2,390
Hours of vigorous activity per week	0.57	[-0.26, 1.40]	2,159
Hours of light activity per week	0.66	[-0.69, 2.02]	2,023
Body mass index	-2.10	[-4.89, 0.69]	2,383
Binary outcomes			
Good health	-0.08+	[-0.17, 0.01]	2,400
Smoking	-0.10	[-0.31, 0.11]	2,393
Alcohol use	0.14	[-0.00, 0.29]	2,399
Binge drinking	0.14	[-0.00, 0.27]	2,366
Heart disease	-0.01	[-0.04, 0.03]	2,401
Hypertension	-0.04	[-0.15, 0.08]	2,401
Diabetes	0.001	[-0.03, 0.03]	2,401

* p <0.05; ** p <0.01. Abbreviations: IV, Instrumental variables; CI, Confidence interval.
Note: Estimates are derived from IV analyses. The endogenous exposure variable was school racial segregation, operationalized as the average dissimilarity index across observed schooling years. The instrument was the proportion of schooling in released districts. We adjusted for 1991 (baseline) school district characteristics of the first observed school district, household income and parental marital status at the first observed schooling year, sex, birth year fixed effects, and state fixed effects of the first observed school district. We also clustered standard errors at the individual level and district level.

Table G. Association of school racial segregation (operationalized as the Black isolation index) with adult cardiovascular risk

Outcome	Estimates	95% CI
Continuous outcomes		
Psychological distress	0.51	[-1.75, 2.77]
Number of cigarettes	-1.38	[-5.62, 2.87]
Hours of vigorous activity per week	0.80	[-0.51, 2.10]
Hours of light activity per week	0.80	[-0.83, 2.43]
Body mass index	-0.28	[-4.71, 4.15]
Binary outcomes		
Good health	-0.14*	[-0.27, -0.02]
Smoking	-0.19	[-0.60, 0.22]
Alcohol use	0.23+	[-0.00, 0.47]
Binge drinking	0.28*	[0.06, 0.51]
Heart disease	-0.01	[-0.06, 0.04]
Hypertension	-0.10	[-0.26, 0.07]
Diabetes	0.01	[-0.05, 0.07]

* p <0.05; ** p <0.01. Abbreviations: IV, Instrumental variables; CI, Confidence interval.
Note: Estimates are derived from IV analyses. The endogenous exposure variable was school racial segregation, operationalized as the average Black isolation index across observed schooling years. The instrument was the proportion of schooling in released districts. We adjusted for 1991 (baseline) school district characteristics of the first observed school district, household income and parental marital status at the first observed schooling year, sex, birth year fixed effects, and state fixed effects of the first observed school district. We also clustered standard errors at the individual level and district level.

Table H. Adjusted P-value for multiple hypothesis testing

Outcomes	OLS		RF		IV	
	Model P-value	Romano-Wolf P-value	Model P-value	Romano-Wolf P-value	Model P-value	Romano-Wolf P-value
Continuous						
Psychological distress	0.0689	0.1782	0.7410	0.9703	0.7401	1.0000
Number of cigarettes	0.9654	1.0000	0.4907	0.8713	0.4995	0.9109
Hours of vigorous activity per week	0.6169	1.0000	0.2497	0.7426	0.2379	0.6733
Hours of light activity per week	0.9908	1.0000	0.3579	0.8416	0.3677	0.8119
Body mass index	0.0603	0.1584	0.8171	0.9703	0.8182	1.0000
Binary						
Good health	0.0001	0.0099	0.0161	0.0495	0.0196	0.0891
Smoking	0.6601	1.0000	0.3187	0.7723	0.3369	0.7822
Alcohol use	0.7367	1.0000	0.0251	0.1089	0.0343	0.0891
Binge drinking	0.0381	0.0990	0.0063	0.0198	0.0083	0.0297
Heart disease	0.0070	0.0198	0.7024	0.9703	0.7062	1.0000
Hypertension	0.6573	1.0000	0.2556	0.7426	0.2661	0.7129
Diabetes	0.5649	1.0000	0.8690	0.9703	0.8684	1.0000

Abbreviations: OLS, Ordinary least squares; RF, Reduced form; IV, Instrumental variables.
Note: Results represent P-values corrected for multiple hypothesis testing using the Romano-Wolf method, accounting clusters by district and individual for a bootstrap sample. “Model P-value” represents results without correction. All OLS, RF, and IV models involved multivariable linear regression. The endogenous exposure variable was school racial segregation, operationalized as the average White-Black dissimilarity index across observed schooling years. For the IV analysis, the instrument was the proportion of schooling in released districts. We adjusted for 1991 (baseline) school district characteristics of the first observed school district, household income and parental marital status at the first observed schooling year, sex, birth year fixed effects, and state fixed effects of the first observed school district. Standard errors were clustered at both district and individual levels.
